# ViCTree: an automated framework for taxonomic classification from protein sequences

**DOI:** 10.1093/bioinformatics/bty099

**Published:** 2018-02-20

**Authors:** Sejal Modha, Anil S Thanki, Susan F Cotmore, Andrew J Davison, Joseph Hughes

**Affiliations:** 1 MRC-University of Glasgow Centre for Virus Research, Glasgow, UK; 2 Earlham Institute, Norwich Research Park, Norwich, UK; 3 Yale University Medical School, New Haven, CT, USA

## Abstract

**Motivation:**

The increasing rate of submission of genetic sequences into public databases is providing a growing resource for classifying the organisms that these sequences represent. To aid viral classification, we have developed ViCTree, which automatically integrates the relevant sets of sequences in NCBI GenBank and transforms them into an interactive maximum likelihood phylogenetic tree that can be updated automatically. ViCTree incorporates ViCTreeView, which is a JavaScript-based visualization tool that enables the tree to be explored interactively in the context of pairwise distance data.

**Results:**

To demonstrate utility, ViCTree was applied to subfamily *Densovirinae* of family *Parvoviridae*. This led to the identification of six new species of insect virus.

**Availability and implementation:**

ViCTree is open-source and can be run on any Linux- or Unix-based computer or cluster. A tutorial, the documentation and the source code are available under a GPL3 license, and can be accessed at http://bioinformatics.cvr.ac.uk/victree_web/.

**Supplementary information:**

Supplementary data are available at *Bioinformatics* online.

## 1 Introduction

The increasing rate at which sequence data are being deposited into public databases is providing a tremendous resource for taxonomic classification throughout biology. Phylogenetic analysis provides a key means of integrating these data and inferring the evolutionary relationships that form the basis of classification. However, preparing datasets for such analyses is often time-consuming, and the phylogenies obtained are typically not easy to update. Consequently, systematic approaches are being developed that automate the various steps involved.

The Ensembl Compara GeneTree pipeline (Vilella *et al.*, 2009) provides a comprehensive gene-orientated phylogenetic resource. It has a powerful analytical backend for classifying genes and gene families on the basis of detecting orthology among the complete genomes available in the Ensembl framework. Automated phylogeny-based classification is also implemented in the mor package (http://www.clarku.edu/faculty/dhibbett/clarkfungaldb/), which has been applied to fungal taxa by aligning 28S rRNA sequences from GenBank and generating a phylogeny that can be updated by a node-based classification approach (Hibbett *et al.*, 2005). The 16S and 18S rRNA sequences can also inform classification, and are employed in tools such as STAP (Wu *et al.*, 2008) and EukRef (http://eukref.org/curation-pipeline-overview/). A more general approach is implemented in PUmPER (Izquierdo-Carrasco *et al.*, 2014), which has been applied to the classification of plants (http://portnoy.iplantcollaborative.org/view/tree/10b17429d13160ac1cd07e30bb42fd9b). However, PUmPER employs PHLAWD (Smith *et al.*, 2009) to collate sequences and build multiple alignments, which in turn relies on GenBank annotations to retrieve nucleotide sequences.

The tools described above were developed for specific types of non-viral organisms and have limited applications to the classification of viruses. Viruses exhibit an enormous range of sequence diversity and cannot be integrated into a tree of life because they lack genes that are universally conserved in other organisms and that therefore may be used for barcoding (e.g. those encoding rRNAs or enzymes such as cytochrome c oxidase subunit I and ribulose-bisphosphate carboxylase). Also, GenBank annotations of viral genes are often not standardized and are thus unreliable for retrieving sequences. Moreover, none of the tools mentioned above presents both pairwise distances and phylogenies. This dual facility is important in viral classification because precise distance thresholds are frequently stipulated as demarcation criteria, and these may vary widely among families and even among genera due to differences in evolutionary rate.

Viruses are classified formally by the International Committee on Taxonomy of Viruses (ICTV; http://www.ictvonline.org/) into three ranks (family, genus and species), and, in some cases, two further ranks (order and subfamily). The huge diversity of viruses has the effect that the criteria used and the relative emphasis placed on each vary widely from family to family. However, sequence-based criteria (typically based on amino acid, rather than nucleotide, sequences) are prominent, and include simple measures of distance and increasingly powerful phylogenetic measures, as in the case of family *Parvoviridae* discussed below (Cotmore *et al.*, 2014). The rapidly increasing volume of viral sequence information and the limitations of existing tools in relation to viruses necessitates the development of automated bioinformatic solutions that are suited specifically to viruses (Simmonds, 2015; Simmonds *et al.*, 2017). At least two tools in this category have been used in viral classification: PASC provides a web-based interface for visualizing distances among automatically aligned sequences (Bao *et al.*, 2014), and DEmARC takes a sophisticated approach to identifying taxonomically significant thresholds in the distribution of distance data (Lauber and Gorbalenya, 2012). These tools were developed for exploring the pairwise distance criteria used to define species and genus boundaries within a taxon. ViPTree, a web-based classification tool, employs a genome-wide similarity method to classify viral sequences, and generates a static viral proteomic tree to illustrate the phylogenetic relationships among the existing sequences available in the GenomeNet/Virus-Host database (Nishimura *et al.*, 2017). Useful as these tools are, none of them presents the results of pairwise distances along with the phylogeny, a feature that is important to viral taxonomists as they transition from using distance-based to phylogeny-based classification methods.

As an aid to integrating GenBank data into taxonomic analyses that can be updated automatically, we present ViCTree, a pipeline that retrieves the relevant viral sequences from GenBank, aligns and clusters them, and generates a maximum likelihood phylogenetic tree combined with distance data. The results are rendered by using ViCTreeView, which is a Javascript-based tool that enables users to visualize and explore distances in the context of the tree. ViCTree is automated so that the phylogenies are synchronized with the GenBank data, and the results are versioned on GitHub. The pipeline is flexible and broadly applicable to examining the phylogenetic relationships that underpin viral taxa.

## 2 Framework

All modules and tools implemented in ViCTree are open-source. The framework is a combination of a Bash shell script for automatically generating multiple sequence alignments and phylogenetic trees, and JavaScript for visualizing and exploring the trees in combination with the underlying distance data.

### 2.1 Phylogeny building

**Fig. 1.**
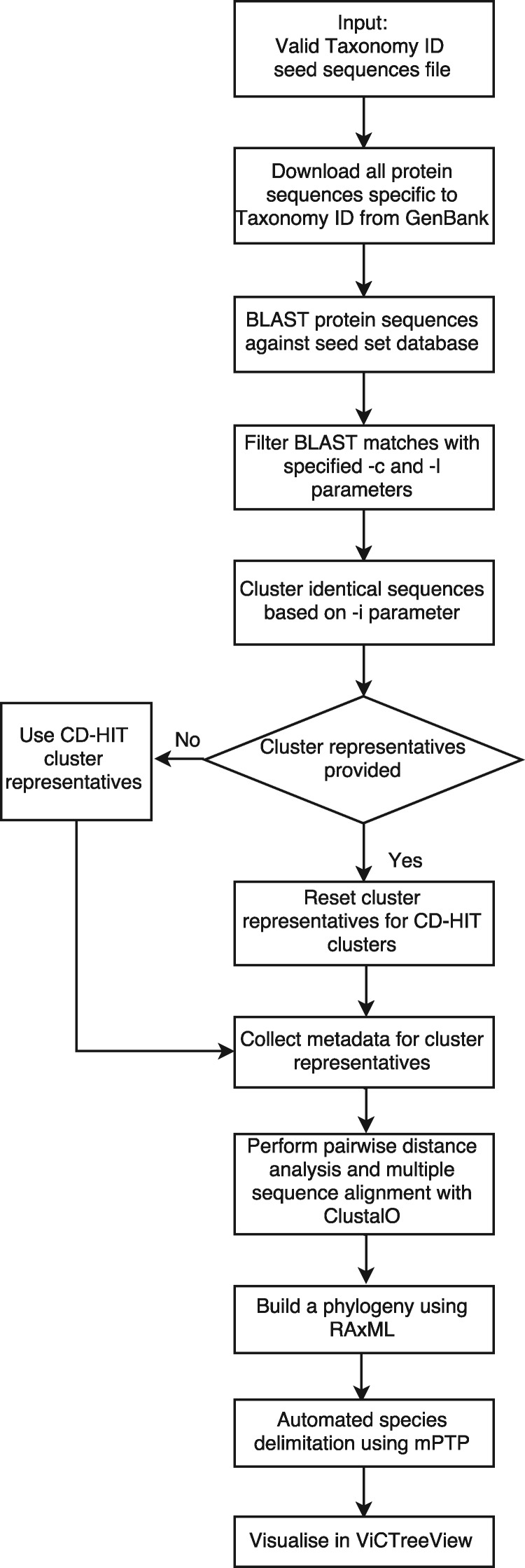
Data processing workflow of the ViCTree pipeline

A curated set of seed protein sequences must be provided that spans the known diversity of a viral taxon. These sequences and the relevant GenBank taxonomic ID (specified at any rank) are submitted to the start of the ViCTree pipeline (Fig. 1), and all available protein sequences that bear the taxonomic ID are automatically downloaded from GenBank. Rather than filtering on the basis of GenBank sequence annotations, which are sometimes incomplete or incorrect, BLAST (Altschul *et al.*, 1990) is used to compare the downloaded sequences with all the seed sequences. Significant matches are extracted from the BLAST output on the basis of user-specified parameters specifying the hit length threshold (the minimum number of amino acid residues in the alignment between the query and subject sequences) and query coverage threshold (the minimum percentage of amino acid residues in the query sequence represented in the alignment). Significant matches are clustered by using CD-HIT (Fu *et al.*, 2012) with a user-specified identity threshold, and a representative sequence from each cluster is selected for downstream analysis in order to reduce the size of the tree to a manageable scale. CD-HIT is used to cluster the sequences below species level to generate a manageable sized phylogeny, with the default clustering threshold set to 0.9. CD-HIT picks the longest sequence as representative by default, and processes the classification of the remaining sequences by comparing them to the representatives. This arrangement has the advantage of not influencing the inter-species or inter-generic relationships. An optional parameter enables the user to provide a list of predefined sequences representing the clusters instead of the default sequences derived by using CD-HIT, thus allowing static tips to be maintained in an expanding phylogeny. A multiple sequence alignment and a distance matrix are generated for the final sequence set by using Clustal Omega (Sievers *et al.*, 2011), and an evolutionary tree is inferred by using RAxML (Stamatakis, 2014) under a user-defined evolutionary model or a default model (PROTGAMMAJTT). The evolutionary tree can then be submitted for automated species delimitation by mPTP (Kapli *et al.*, 2017).

The output files include the tree in Newick format, the alignment in Fasta format, a distance matrix, a comma-separated list of clustered sequences, and a metadata file with GenBank accession numbers and taxonomic names (species and genus) if known. These files are stored on GitHub in a predefined directory structure, thus allowing previous versions of the alignment and tree to be retrieved and enabling changes to be tracked over time. After an initial setup stage for the viral group of interest, the phylogeny can be updated with little or no manual intervention.

### 2.2 Tree visualization

ViCTreeView was inspired by VEG’s phylotree (https://github.com/veg/phylotree.js) and enables the tree to be visualized. In ViCTreeView, maximum likelihood phylogenetic trees are rendered directly from the GitHub repository and visualized as a phylogram with bootstrap values. It is possible to visualize the tree in ultrametric representation instead of a phylogram. Different phylogenetic tree instances available in the GitHub repository can be browsed and visualized using by using the example menu. Attributes to increase and decrease distances between branches and a zooming function styled after Google maps are implemented in ViCTreeView, thus enabling users to explore specific parts of the phylogeny in detail. This interactive web tool facilitates an integrated dynamic visualization of the tree and the distance data represented as percentages. When a user-defined distance threshold is specified, sequences that fall within it are highlighted in clusters of different colours. This enables users to study and explore the sequences that generate new clusters when a specific pairwise distance criterion is applied. The highlighted versions of the tree with user-defined thresholds are also available for downloading in SVG and PNG formats. The automated phylogeny generated within ViCTree is mid-point rooted and can be re-rooted to any nodes in the phylogeny. Specific branches can be expanded and collapsed in order to explore large phylogenetic trees in modular fashion. In addition, various features are included to allow manipulation of the tree, including options for labelling the tips by GenBank accession number, taxonomic ID, species name or genus name. These options, along with the alignment files from GitHub repositories, enable users to download the tree files in a specific format, and also facilitate easy incorporation of data for newly discovered viruses into taxonomic proposals. Links are also provided to the NCBI genomes page for representative sequences and to the NCBI proteins page for the representative and non-representative protein sequences clustered in the phylogeny.

ViCTree can be run on any Linux/Unix or OSX Apple computer. It was tested on an Apple iMac with a 4 GHz Intel Core i7 processor and 32 GB RAM.

## 3 Results

### 3.1 Case study

The example framework (http://bioinformatics.cvr.ac.uk/victree/) is setup for subfamilies *Densovirinae* and *Parvovirinae* of family *Parvoviridae* and for family *Herpesviridae*, and is updated monthly on an automatic schedule. To illustrate the application of ViCTree, we present the results for subfamily *Densovirinae*.

Within the family *Parvoviridae*, viruses that infect invertebrates and vertebrates are classified into the two subfamilies *Densovirinae* and *Parvovirinae*, respectively. This division is strongly supported by the protein sequence-based phylogeny of viral non-structural protein 1 (NS1) (Cotmore *et al.*, 2014). All viruses within the same species are required to be at least 85% identical to each other in this protein, and at least 15% different from viruses in other species. Viruses within the same genus are required to be monophyletic and to encode NS1 proteins that are at least 30% identical to each other. These demarcation criteria were applied to an analysis of subfamily *Densovirinae* carried out by using ViCTree. The analysis took 552 min 34.983 s real time, 1019 min 38.773 s user time and 29 min 32.492 s system time.

The analysis of subfamily *Densovirinae* runs automatically every month as a Cron job. In June 2017, 916 protein sequences available under the relevant taxonomic ID (40120) were downloaded automatically from GenBank. A subset of 21 NS1 protein sequences was used as the seed set in a BLAST-based similarity search of all downloaded protein sequences. A subset of 187 sequences was generated when hit length and query coverage thresholds of 100 and 50, respectively, were applied to filter the BLAST output. These sequences grouped into 103 distinct clusters when a CD-HIT clustering threshold of 1.0 was applied. Distance analysis and multiple sequence alignment were performed on the 103 representatives of these clusters, and taxonomic and accession metadata were collected from GenBank. A phylogeny was built for the aligned sequences, and the tree file was submitted with metadata and distance matrix files to ViCTreeView for visualization.

ViCTree identified all previously classified species and genera in subfamily *Densovirinae* (Cotmore *et al.*, 2014), including members of genus *Ambidensovirus* that encode the NS1 protein on the opposite strand from members of the other genera. This success was due to the breadth of diversity in the seed set and to the use of protein sequences in conducting the BLAST search. The analysis identified six new species (Table 1), which were recognized subsequently by the ICTV (Adams *et al.*, 2017b) on the basis of proposals made by the ICTV *Parvoviridae* Study Group. Although ViCTree is not a dedicated taxonomic misclassification identification tool such as SATIVA (Kozlov *et al.*, 2016) it is able to identify misclassified sequences. Thus, the misclassification of an isolate of Helicoverpa armigera densovirus (GenBank accession number JQ894784) in the NCBI taxonomy was readily identified. This virus is described as being a member of genus *Iteradensovirus*, but in fact belongs to genus *Ambidensovirus* (species *Lepidopteran iteradensovirus 5*).

**Table 1.** New species identified in subfamily *Densovirinae* by using ViCTree

Name of new species	Representative isolate	Genus
*Asteroid ambidensovirus 1*	Sea star-associated densovirus	*Ambidensovirus*
*Decapod ambidensovirus 1*	Cherax quadricarinatus densovirus	*Ambidensovirus*
*Hemipteran ambidensovirus 2*	Dysaphis plantaginea densovirus 1	*Ambidensovirus*
*Hemipteran ambidensovirus 3*	Myzus persicae densovirus 1	*Ambidensovirus*
*Hymenopteran ambidensovirus 1*	Solenopsis invicta densovirus	*Ambidensovirus*
*Orthopteran densovirus 1*	Acheta domestica mini ambidensovirus	*Unassigned*

*Source*: https://talk.ictvonline.org/ICTV/proposals/2016.003a, bD.A.v1.Densovirinae_6sp.pdf

The in-built automated species delimitation using mPTP had identified a total of 25 species from the phylogeny, of which 18 were consistent with the current ICTV classification of subfamily Densovirinae. The automated species delimitation combined *Decapod ambidensovirus 1* and *Asteroid ambidensovirus 1* into a species cluster, and *Lepidopteran iteradensovirus 1*, *Lepidopteran iteradensovirus 2* and *Lepidopteran iteradensovirus 4* into another species cluster. It also identified an additional six new species that are not currently recognized by the ICTV (Supplementary Appendix S1). Some of these species are not yet defined as new species by the ICTV as the sequences may be from incomplete genome sequences or may lack multiple sequences from the same species, both of which are requirements for the assignment of new species.

### 3.2 Evaluation of accuracy

**Fig. 2.**
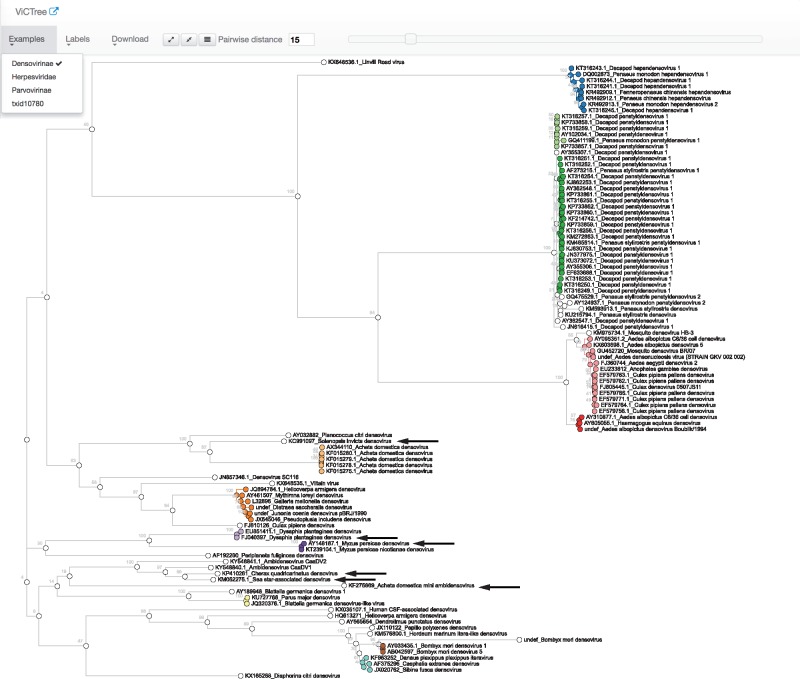
Phylogenetic tree for subfamily *Densovirinae* based on the NS1 protein and visualized in ViCTreeView. Sequences that fall within the 15% pairwise distance criterion are indicated as distinct clusters in different colours. Black arrows indicate new species identified using ViCTree

**Fig. 3.**
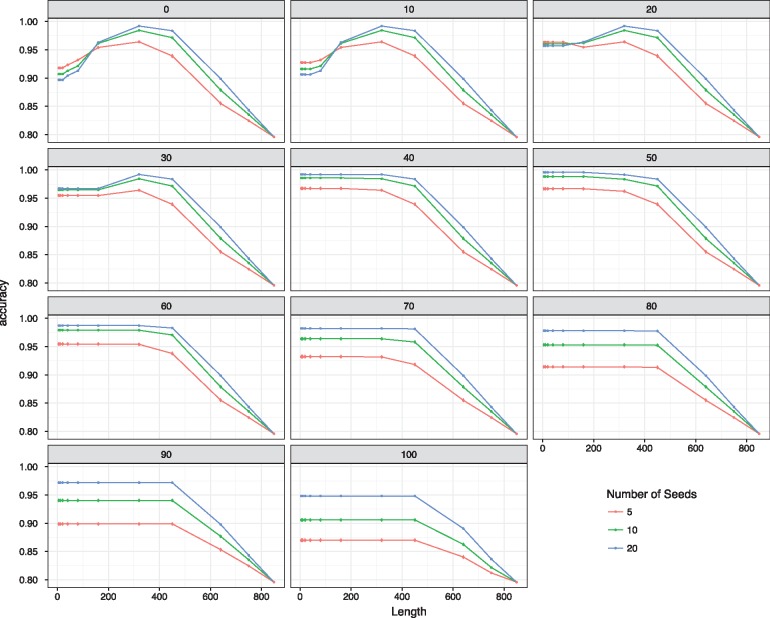
Accuracy (*y*-axis) of ViCTree in relation to BLAST query coverage (0–100), BLAST hit length (0–849 amino acid residues) and number of seed sequences (5–20)

The accuracy of the ViCTree was tested by determining the proportion of recognized species that it was capable of identifying in subfamily *Densovirinae* (Fig. 2). Three parameters were varied: the number of seed sequences (sets of 5, 10 or 20 randomly selected sequences), the hit length threshold, and the query coverage threshold (Fig. 3). Accuracy increased with the number of seed sequences, and was >95% for all seed sequence sets at a hit length of <400 and a query coverage of <60. Accuracy was compromised by reducing these values, due to increasing numbers of false positives. Hit length and query coverage thresholds of 100 and 50, respectively, were found to be optimal for subfamily *Densovirinae*.

## 4 Discussion

ViCTree is an integrated, automated pipeline for assisting taxonomic classification in an era in which genomic and metagenomic data are being actively accommodated by the ICTV (Adams *et al.*, 2017a; Simmonds, 2015; Simmonds *et al.*, 2017). It is capable of supporting the identification of novel viral species and pinpointing taxonomic errors in public databases. Its automated approach to finding the best reference sequences to represent a viral family or subfamily provides a useful tool for virologists. It implements GitHub-based versioning of alignments and phylogenies of any size, thus allowing users to monitor taxonomic developments incrementally. The built-in visualization tool (ViCTreeView) enables phylogenies to be explored interactively in a web browser. These features will contribute to the establishment and dissemination of standardized phylogenetic and taxonomic data within the virology community.

The initial setup of ViCTree for a taxonomic group requires several optimization steps, which include setting the thresholds for seed sequences, CD-HIT clustering, and BLAST hit length and query coverage. These parameters were shown to be accurate in the case study of subfamily *Densovirinae*, but will need to be improved iteratively as the taxonomy expands. They will differ for other viral taxa; for example, a single DNA polymerase protein seed sequence was sufficient to identify all species in family *Herpesviridae*. In a wider context, the criteria used to classify viruses vary greatly from family to family, and the flexibility of ViCTree allows appropriate thresholds to be explored interactively. Accuracy determination for a specific viral group of interests is deemed to be an iterative process, as classification parameters depend on the new sequences identified and incorporated into the seed set used as a starting point for ViCTree analysis. The ViCTree GitHub repository provides scripts that enable users to identify optimal BLAST and seed set parameters to study a viral taxonomic group using ViCTree. Novel sequences that are yet to be submitted to GenBank can also be explored using the ViCTree framework, as ViCTree allows the incorporation of these sequences by adding them to the seed sequence set.

Since ViCTree is a pipeline that integrates a number of existing tools, it will suffer from their limitations. Clustal Omega was incorporated because it can align large numbers of protein sequences quickly and accurately (Sievers *et al.*, 2011). However, like other progressive algorithms such as CLUSTAL W (Thompson *et al.*, 1994), MAFFT (Katoh *et al.*, 2002; Katoh and Standley, 2013), MUSCLE (Edgar, 2004) and T-COFFEE (Di Tommaso *et al.*, 2011), it may suffer from shortcomings in the handling of insertions and deletions. Other phylogeny-aware methods that have been developed for accurately aligning closely related sequences, such as PRANK (Löytynoja and Goldman, 2005, 2008), are less susceptible to these problems and may improve ViCTree in future, particularly for delimiting genotypes within viral species.

ViCTree adds to a growing number of sequence-based tools that are designed to inform viral classification specifically or that may prove to be adaptable from other areas of biology. Pairwise distance criteria currently being used across the field of viral taxonomy does not provide an objective approach to classify groups of viruses and other methods such as GMYC and PTP/mPTP should be explored further in the context of speciation and identification of novel viral groups. The current version of ViCTree uses protein sequences as input because amino acid sequences are typically used to distinguish taxa from the level of family (or sometimes order) down to species (e.g. *Parvoviridae* and *Herpesviridae*). Although ViCTree was developed with viral classification in mind, it could be used to explore the evolution of any protein.

## Supplementary Material

Supplementary Data

## References

[bty099-B1] Adams M.J. et al (2017a) 50 years of the International Committee on Taxonomy of Viruses: progress and prospects. Arch. Virol., 162, 1441–1446. 28078475 10.1007/s00705-016-3215-y

[bty099-B2] Adams M.J. et al (2017b) Changes to taxonomy and the International Code of Virus Classification and Nomenclature ratified by the International Committee on Taxonomy of Viruses (2017). Arch. Virol., 162, 2505–2538. 28434098 10.1007/s00705-017-3358-5

[bty099-B3] Altschul S.F. et al (1990) Basic local alignment search tool. J. Mol. Biol., 215, 403–410. 2231712 10.1016/S0022-2836(05)80360-2

[bty099-B4] Bao Y. et al (2014) Improvements to pairwise sequence comparison (PASC): a genome-based web tool for virus classification. Arch. Virol., 159, 3293–3304. 25119676 10.1007/s00705-014-2197-x PMC4221606

[bty099-B5] Cotmore S.F. et al (2014) The family Parvoviridae. Arch. Virol., 159, 1239–1247. 24212889 10.1007/s00705-013-1914-1 PMC4013247

[bty099-B6] Edgar R.C. (2004) MUSCLE: multiple sequence alignment with high accuracy and high throughput. Nucleic Acids Res., 32, 1792–1797. 15034147 10.1093/nar/gkh340 PMC390337

[bty099-B7] Fu L. et al (2012) CD-HIT: accelerated for clustering the next-generation sequencing data. Bioinformatics, 28, 3150–3152. 23060610 10.1093/bioinformatics/bts565 PMC3516142

[bty099-B8] Hibbett D.S. et al (2005) Points of View Automated Phylogenetic Taxonomy: an Example in the Homobasidiomycetes (Mushroom-Forming Fungi). Syst. Biol., 54, 660–668. 16126660 10.1080/10635150590947104

[bty099-B9] Izquierdo-Carrasco F. et al (2014) PUmPER: phylogenies updated perpetually. Bioinformatics, 30, 1476–1477. 24478338 10.1093/bioinformatics/btu053 PMC4016711

[bty099-B10] Kapli P. et al (2017) Multi-rate Poisson Tree Processes for single-locus species delimitation under Maximum Likelihood and Markov Chain Monte Carlo. Bioinformatics, 33, btx025. 10.1093/bioinformatics/btx025 PMC5447239 28108445

[bty099-B11] Katoh K. et al (2002) MAFFT: a novel method for rapid multiple sequence alignment based on fast Fourier transform. Nucleic Acids Res., 30, 3059–3066. 12136088 10.1093/nar/gkf436 PMC135756

[bty099-B12] Katoh K., Standley D.M. (2013) MAFFT Multiple Sequence Alignment Software Version 7: improvements in Performance and Usability. Mol. Biol. Evol., 30, 772–780. 23329690 10.1093/molbev/mst010 PMC3603318

[bty099-B13] Kozlov A.M. et al (2016) Phylogeny-aware identification and correction of taxonomically mislabeled sequences. Nucleic Acids Res., 44, 5022–5033. 27166378 10.1093/nar/gkw396 PMC4914121

[bty099-B14] Lauber C., Gorbalenya A.E. (2012) Partitioning the genetic diversity of a virus family: approach and evaluation through a case study of picornaviruses. J. Virol., 86, 3890–3904. 22278230 10.1128/JVI.07173-11 PMC3302503

[bty099-B15] Löytynoja A., Goldman N. (2008) Phylogeny-aware gap placement prevents errors in sequence alignment and evolutionary analysis. Science, 320, 1632–1635. 18566285 10.1126/science.1158395

[bty099-B16] Löytynoja A., Goldman N. (2005) An algorithm for progressive multiple alignment of sequences with insertions. Proc. Natl. Acad. Sci. USA, 102, 10557–10562. 16000407 10.1073/pnas.0409137102 PMC1180752

[bty099-B17] Nishimura Y. et al (2017) ViPTree: the viral proteomic tree server. Bioinformatics., 33, 2379–2380. 28379287 10.1093/bioinformatics/btx157

[bty099-B18] Sievers F. et al (2011) Fast, scalable generation of high-quality protein multiple sequence alignments using Clustal Omega. Mol. Syst. Biol., 7, 539. 21988835 10.1038/msb.2011.75 PMC3261699

[bty099-B19] Simmonds P. et al (2017) Consensus statement: virus taxonomy in the age of metagenomics. Nat. Rev. Microbiol., 15, 161–168. 28134265 10.1038/nrmicro.2016.177

[bty099-B20] Simmonds P. (2015) Methods for virus classification and the challenge of incorporating metagenomic sequence data. J. Gen. Virol., 96, 1193–1206. 26068186 10.1099/jgv.0.000016

[bty099-B21] Smith S.A. et al (2009) Mega-phylogeny approach for comparative biology: an alternative to supertree and supermatrix approaches. BMC Evol. Biol., 9, 37. 19210768 10.1186/1471-2148-9-37 PMC2645364

[bty099-B22] Stamatakis A. (2014) RAxML version 8: a tool for phylogenetic analysis and post-analysis of large phylogenies. Bioinformatics, 30, 1312–1313. 24451623 10.1093/bioinformatics/btu033 PMC3998144

[bty099-B23] Thompson J.D. et al (1994) CLUSTAL W: improving the sensitivity of progressive multiple sequence alignment through sequence weighting, position-specific gap penalties and weight matrix choice. Nucleic Acids Res., 22, 4673–4680. 7984417 10.1093/nar/22.22.4673 PMC308517

[bty099-B24] Di Tommaso P. et al (2011) T-Coffee: a web server for the multiple sequence alignment of protein and RNA sequences using structural information and homology extension. Nucleic Acids Res., 39, W13–W17. 21558174 10.1093/nar/gkr245 PMC3125728

[bty099-B25] Vilella A.J. et al (2009) EnsemblCompara GeneTrees: complete, duplication-aware phylogenetic trees in vertebrates. Genome Res., 19, 327–335. 19029536 10.1101/gr.073585.107 PMC2652215

[bty099-B26] Wu D. et al (2008) An Automated Phylogenetic Tree-Based Small Subunit rRNA Taxonomy and Alignment Pipeline (STAP). PLoS One, 3, e2566. 18596968 10.1371/journal.pone.0002566 PMC2432038

